# Supplementary material for: Norepinephrine transporter defects lead to sympathetic hyperactivity in Familial Dysautonomia models
Source: Nat Commun. 2022 Nov 17;13:7032. doi: 10.1038/s41467-022-34811-7 (PMC9671909; doi:10.1038/s41467-022-34811-7)
Supplement: Supplementary file 1 — Supplementary Information [file 41467_2022_34811_MOESM1_ESM.pdf]

Supplementary figures

Supplementary figure 1 (related to figure 1). Sympathetic neuron differentiation protocol improvements.

(**a**) Schematic illustration of the updated symN differentiation steps and experimental time points. Aphi=aphidicolin. (Below) day 10 NC FACS gating strategy. (**b**) Sympathetic lineage markers are verified in day 14-20 symNs using RT-qPCR. N=(*ASCL1*/*PHO2B*: 7, *PRPH*/*HAND2*: 6, *GATA3*: 3) biological replicates. Error bar represents the SEM. (**c**) HOX code is also examined in day 14-20 symNs and trunk identity is defined (i.e. *HOXB5*$^{+}$/*B7*$^{+}$/*C9*$^{+}$). N=(*HOXB2*/*HOXC9*: 9, *HOXB5*/*HOXB7*: 3, *HOXA10*: 4) biological replicates. Error bar represents the SEM. (**d**) SymNs form clusters with axon extension by day 35. (**d'**) PHOX2B^{+} symNs can be identified on day 30 using the eGFP::PHOX2B-H9 line (Oh, 2016). Scale bar in **d'** represents 50 µm. (**e-g**) Expression of genes important for symN activation (**e**, N=(*CHRNA3*: 4, *CHRNB4*/*VMAT1*: 5) biological replicates), NE autoregulation (**f**, N=(*ADRA2A*/*NET*: 10, *ADR2B*: 7) biological replicates) and NE production (**g**, N=(*TH*: 10, *AAAD*/*MAO-A*: 7, *DBH*: 8) biological replicates) are also confirmed by RT-qPCR in day 20-30 symNs. Error bar represents the SEM. (**h**) Immunostaining shows autoregulatory markers (ARs and NET, green) and NE (green) in day 30 symNs. (**i-j**) Cryopreservation. Cell survival of day 10 frozen NCCs at high ($8x10^6$/vial) or low ($4x10^6$/vial) density from ESC or iPSC origins are calculated after thaw (**i**). n=3 biological replicates. Error bar represents the SEM. Scale bars represent 100 µm. Control symN activities with or without freezing are

compared on day 30 using MEA (**j**). n=3 biological replicates. Two-tailed Student's t-test. Error bar represents the SEM. Source data are provided as a Source Data file.

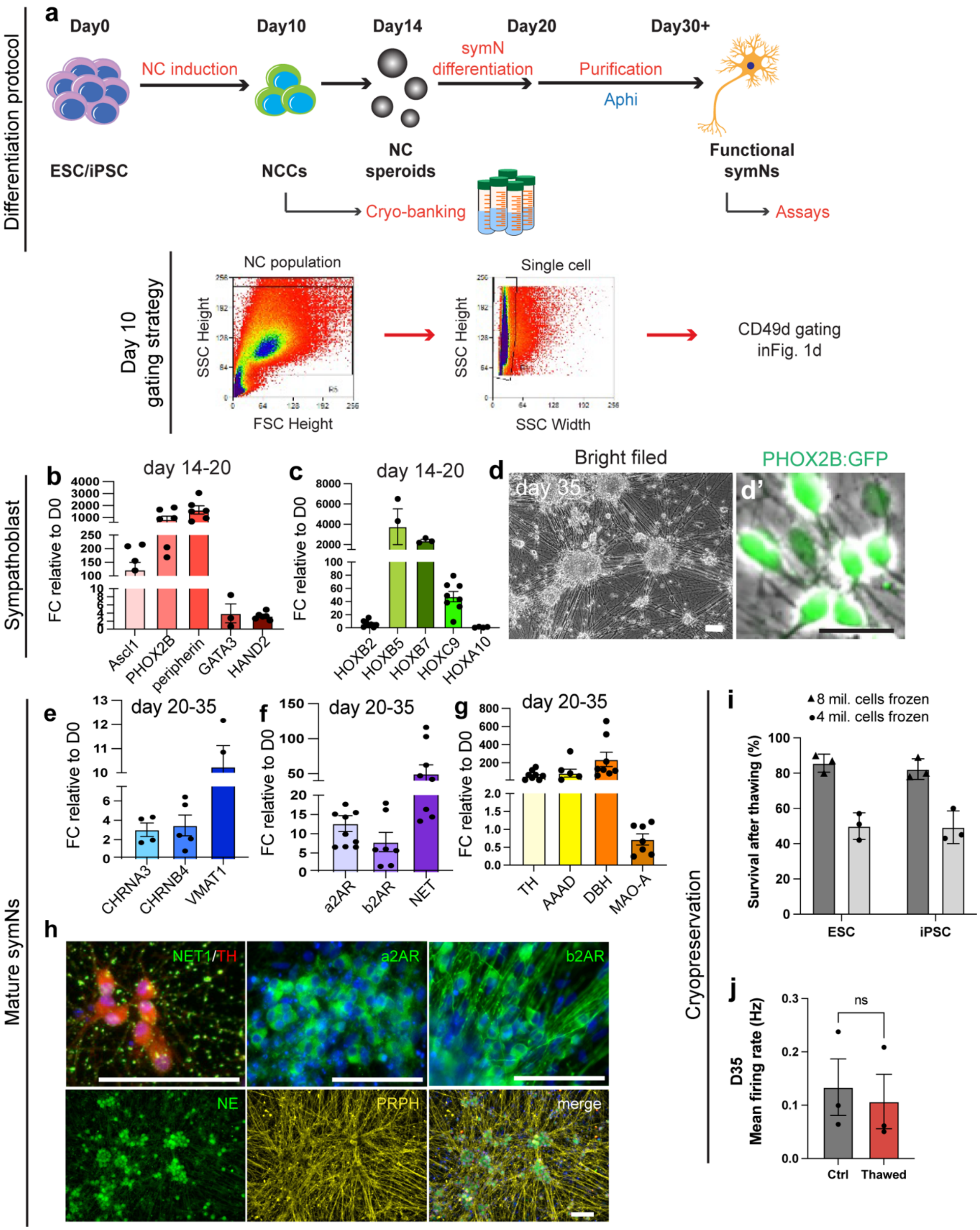

Supplementary figure 2 (related to figure 1). Sympathetic neuron purity and functionality.

(**a**) Aphidicoline (Aphi) treatment (0.5 μM) on day 20 for ten days eliminates most non-neural cells in symN differentiation. Quantification of $TH^+/DAPI^+$ cell percentage shows increased TH^+ symN number after Aphi treatment, while neural specification calculated as $TH^+/PRPH^+$ cell percentage is always high. N=4-9 biological replicates. Two-tailed multiple unpaired Student's t-test. Error bar represents the SEM. ****$p<0.0001$. (**b**) SymNs are stained for TH (green) and PRPH (red), and non-neural cells are calculated as $DAPI^+$ only cells. (**c**) Immunostaining of day 30 symNs ($PRPH^+$ in yellow) shows eliminated proliferating cells ($Ki67^+$ in red) and minimal remaining NCCs ($SOX10^+$ in green) after Aphi treatment. (**d**) Representative spike rate heatmaps show firing of day 30 symNs. Day 0 hPSCs are also measured as negative controls. (**e**) Quantification of spike rate indicates neural functionality, and burst rates indicates neural maturity. N=9 biological replicates. Error bar represents the SEM. (**f**) ChAT (green) and PRPH (red) immunostaining of hPSC-symNs and hPSC-SNs. (**g**) Comparisons of NE and Ach release from symNs by ELISA. N=3 biological replicates. Two-tailed Student's t-test. Error bar represents the SEM. ****$p<0.0001$. Scale bars represent 200 μm. Source data are provided as a Source Data file.

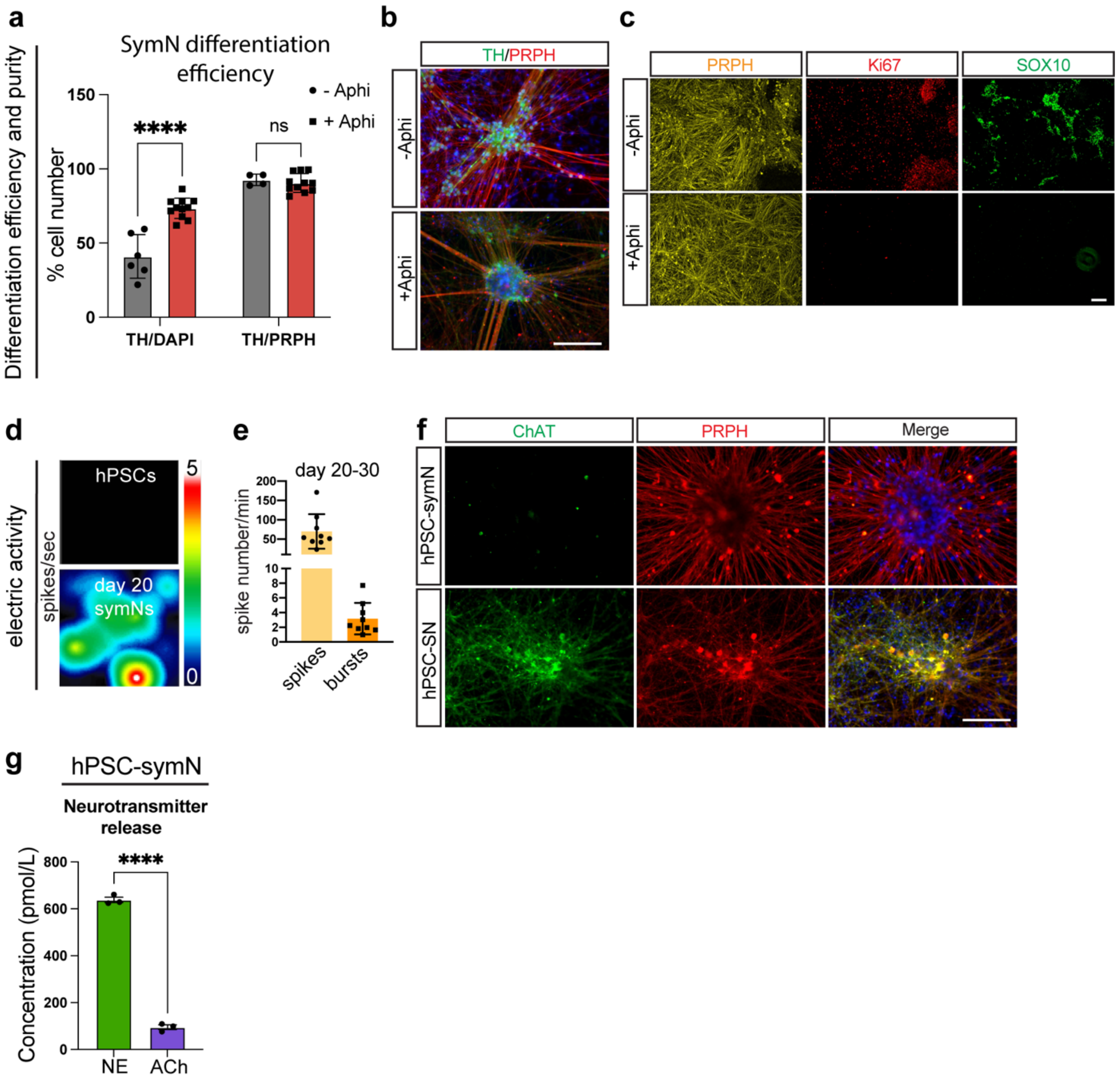
a
SymN differentiation efficiency
Differentiation efficiency and purity
% cell number
- Aphi
+ Aphi
****
ns
TH/DAPI
TH/PRPH
b
TH/PRPH
-Aphi
+Aphi
c
PRPH
Ki67
SOX10
-Aphi
+Aphi
d
electric activity
spikes/sec
hPSCs
day 20 symNs
e
day 20-30
spike number/min
spikes
bursts
f
ChAT
PRPH
Merge
hPSC-symN
hPSC-SN
g
hPSC-symN
Neurotransmitter release
Concentration (pmol/L)
****
NE
ACh

Supplementary figure 3 (related to figure 1). Confirmation of pluripotency in human pluripotent stem cell lines.

(**a**) Immunostaining for NANOG (green) and OCT4 (red) shows similar patterns among hPSCs. Bright field pictures (bottom row) confirm that hPSCs are at an ideal status for symN differentiation. Scale bars represent 200 μm.

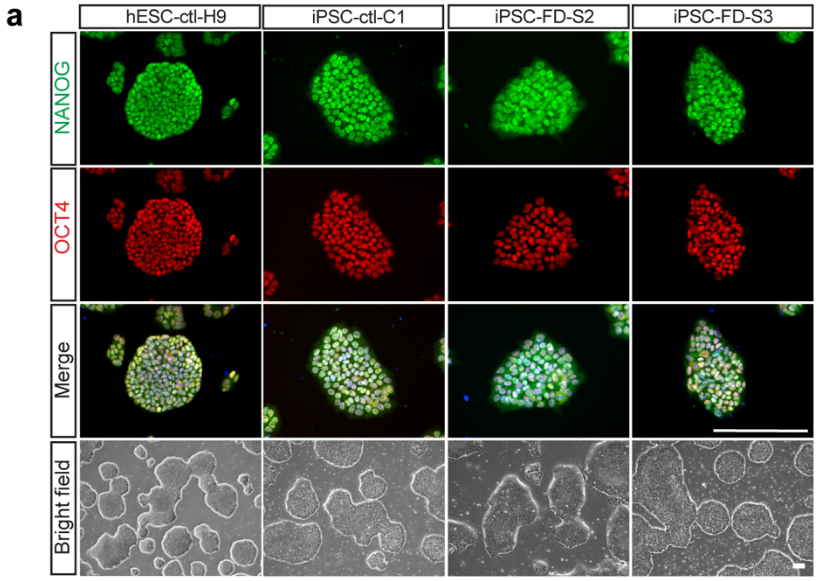

Supplementary figure 4 (related to figure 1). Characterization of day 10 neural crest cells (NCCs).

(**a**) Day 10 NCC cell numbers in control and FD cultures. N=(ctrl: 21, FD: 14) biological replicates. Two-tailed Student's t-test. Error bar represents the SEM. (**b**) Day 10 NCCs immunostained for SOX10 (green) and AP2a (red) in control and FD. (**c**) RT-qPCR alanysis of day 10 control NCCs for contaminating cells. N=6 biological replicates. Error bar represents the SEM. Scale bar represents 200 μm. In a, data from hESC-ctrl-H9 and iPSC-ctrl-C1 are pooled as control; data from iPSC-FD-S2 and iPSC-FD-S3 are pooled as FD. Source data are provided as a Source Data file.

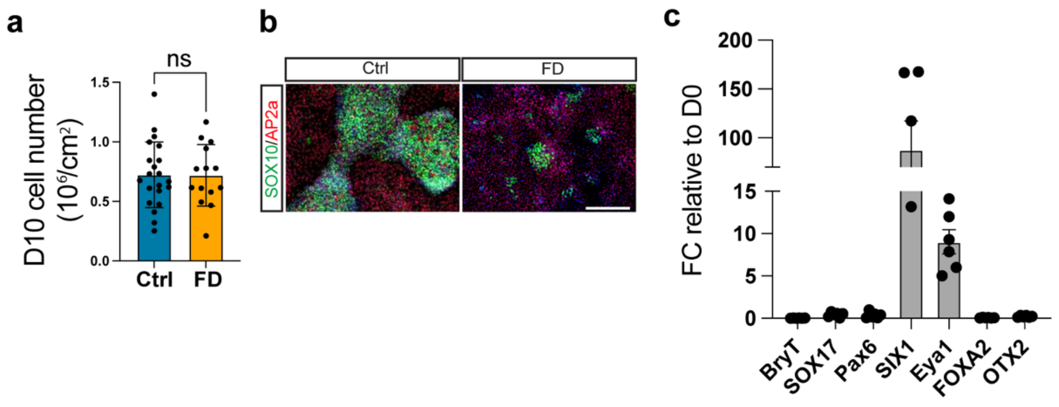

Supplementary figure 5 (related to figure 2). Cardiomyocyte (CM)-sympathetic neuron co-culture system.

(**a**) Bright field picture shows beating CMs on day 7. See also Sup. Movie 1. (**b**) CM beating can be measured by MEA as early as day 10. (**c**) CM gene expression is confirmed using RT-qPCR on day 15. N=(*CTNT*: 5, *NKX2.5*: 5) biological replicates. Error bar represents the SEM. (**d**) d’, cartoon of anatomy of CM-symN junction. In d’’-d’’’’, one week after the co-culture initiation, cells are stained for CM marker α-Actinin (green) and symN marker PRPH (red). White arrows indicates the nodal structures of neural cardiac junctions where symNs innervate CM (as shown in d'). Scale bars in d’’’ and d’’’’ represent 50 μm. In d”, symN axons (red) are attracted and grow deeply into the CM (gray). Physical contacts between symNs and CMs are shown by synaptophysin staining (SYP, green). (**e**) MEA measurements show the CM beating rate with multiple culture conditions. N=(CM: 4, rest: 3) biological replicates. One-way ANOVA followed by Tukey’s multiple comparisons. Error bars represent the SEM. *P<0.05, **p<0.01, ***P<0.001. Scale bars represent 200 μm. Source data are provided as a Source Data file.

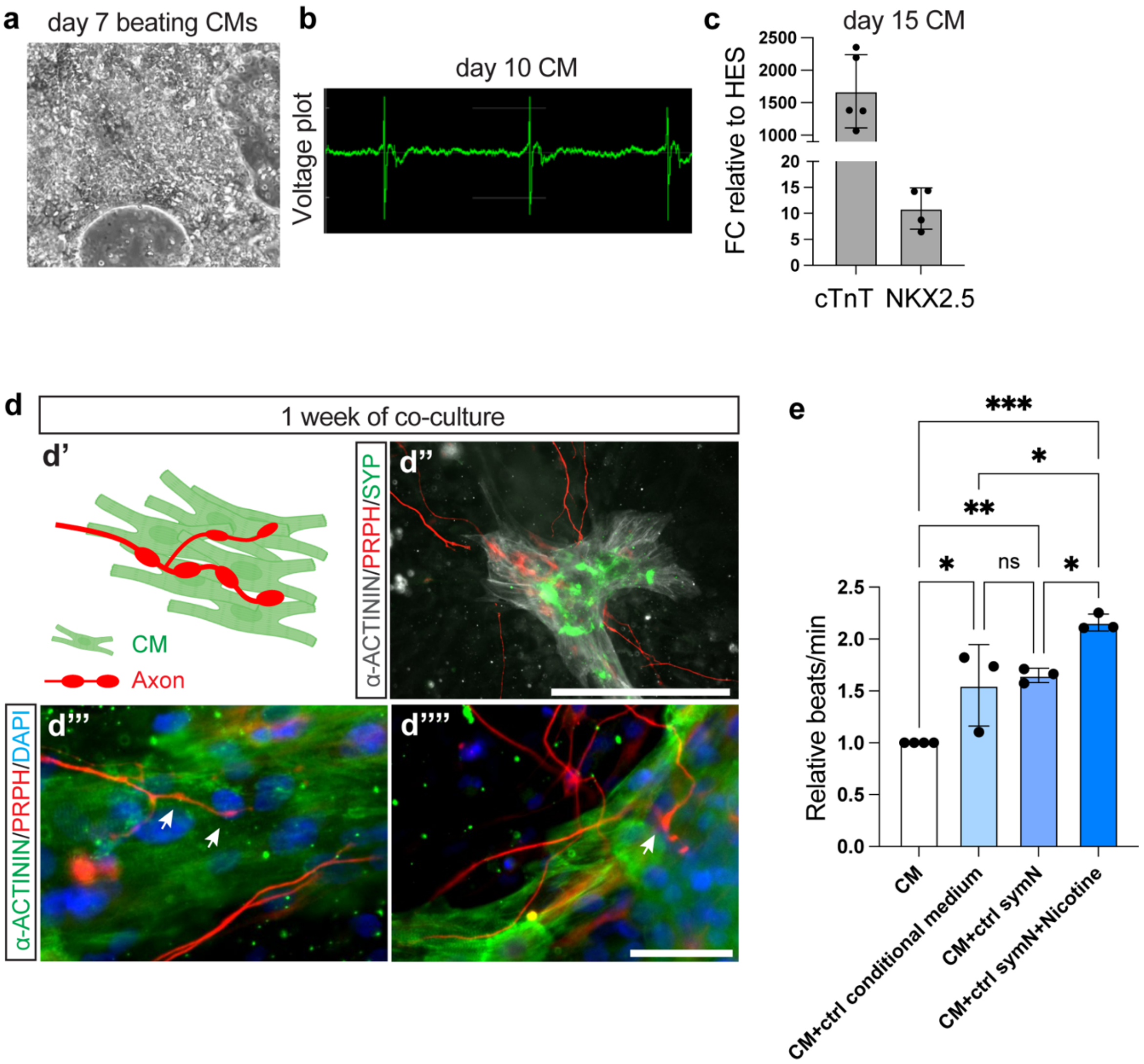
a
day 7 beating CMs
b
day 10 CM
Voltage plot
c
day 15 CM
FC relative to HES
2500
2000
1500
1000
20
15
10
5
0
cTnT
NKX2.5
d
1 week of co-culture
d’
CM
Axon
d”
α-ACTININ/PRPH/SYP
d’’’
α-ACTININ/PRPH/DAPI
d’’’’
e
***
*
**
*
ns
*
Relative beats/min
2.5
2.0
1.5
1.0
0.5
0.0
CM
CM+ctrl conditional medium
CM+ctrl symN
CM+ctrl symN+Nicotine

Supplementary figure 6 (related to figure 3). Familial Dysautonomia mouse model

(**a**) Cartoon shows *Wnt1/Sox10*-cre; elp1$^{LoxP/LoxP}$ lineage-specific CKO designs. (**b**) Picture demonstrates the identification of superior cervical ganglia (SCG) in E14.5 embryos. (**c**) Cultured WT and CKO SCGs from *Wnt1*-Cre; elp1$^{LoxP/LoxP}$ mouse line after DIV10. Red rectangles highlight the neural bodies and green rectangles show the neurite morphologies. Scale bar represents 50 µm.

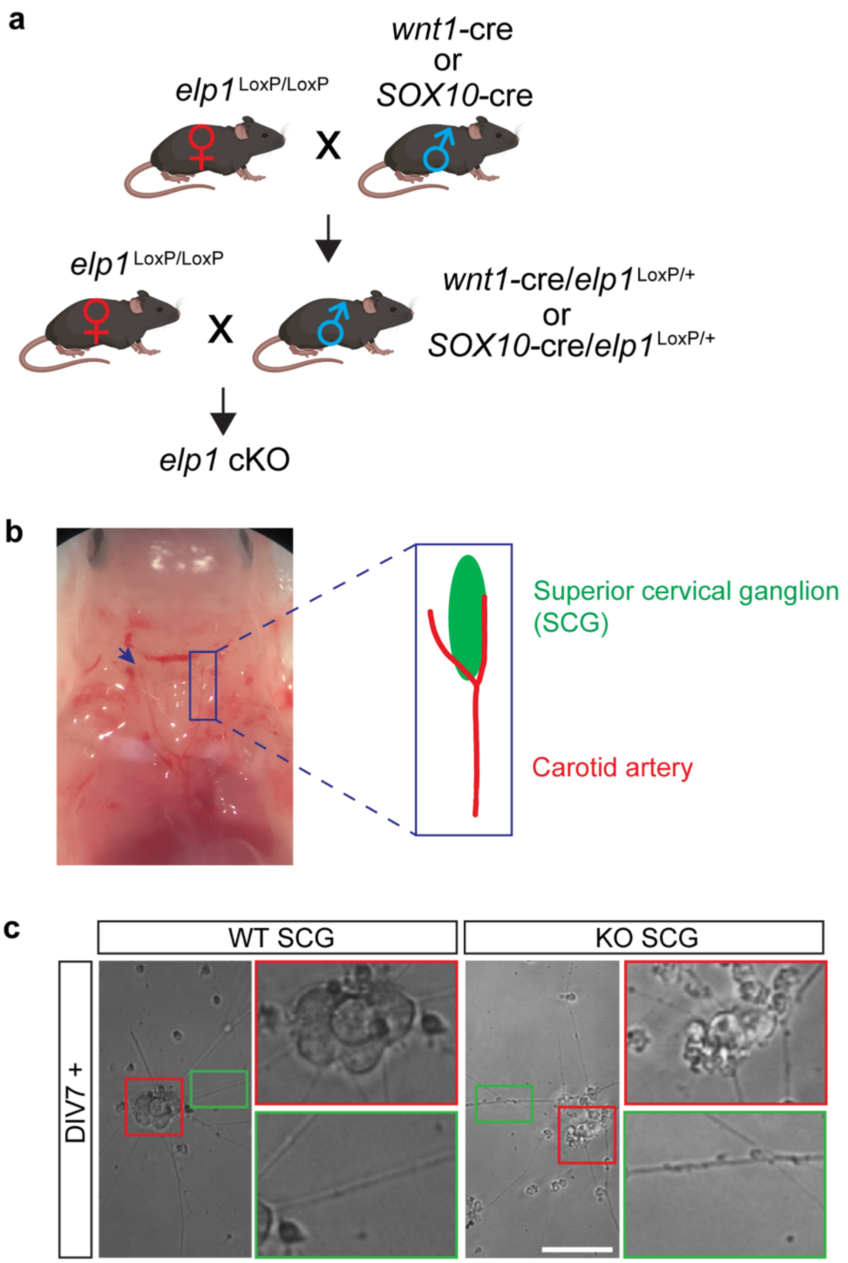

a
wnt1-cre
or
SOX10-cre
elp1 LoxP/LoxP
X
elp1 LoxP/LoxP
X
wnt1-cre/elp1 LoxP/+
or
SOX10-cre/elp1 LoxP/+
elp1 cKO
b
Superior cervical ganglion
(SCG)
Carotid artery
c
WT SCG
KO SCG
DIV7 +

Supplementary figure 7 (related to Figure 4). Effects of the *ELP1* mutation on hyperactivity

(**a**) MEA measurements on symNs derived from iPSC-rescued-T6 still shows hyperactivity compared to hESC-ctrl-H9 (same data as in Fig. 2b for ctrl). N=(D20-H9: 9, T6: 4; D25-H9: 9, T6: 4; D30-H9: 14, T6: 5; D35-H9: 10, T6: 8; D40-H9: 7, T6: 3; D45-H9: 3, T6: 3; D50-H9: 3, T6: 4; D55-H9: 5, T6: 4; D60-H9: 6, T6: 5) biological replicates. Two-way ANOVA followed by Šídák multiple comparisons. Error bar represents the SEM. *$p<0.05$, ***$P<0.001$. (Right) MEA shows increased weighted mean firing rate in FD and FD-rescued symNs. N=(ctrl/FD: 12, T6: 6) biological replicates. One-way ANOVA followed by Tukey's multiple comparisons. ****$P<0.0001$. (**b**) Immunostaining for NANOG (green) and OCT4 (red) shows similar patterns among hPSCs. Bright field pictures (bottom row) confirm that hPSCs are at an ideal status for differentiation. Scale bars represent 200 µm. (**c**) MEA measurements on symNs derived from hESC-ctrl-652 and iPSC-carrier-A1 show similar patterns compared to hESC-ctrl-H9 (same data as in Fig. 2b for ctrl). N=(D20-H9: 9, 652: 3, A1: 5; D25-H9: 9, 652: 3, A1: 5; D30-H9: 14, 652: 3, A1: 10; D35-H9: 10, 652: 3, A1: 5; D40-H9: 7, 652: 6, A1: 4; D45-H9: 3, 652: 4, A1: 4; D50-H9: 3, 652: 6, A1: 3; D55-H9: 5, 652: 7, A1: 4; D60-H9: 6, 652: 8, A1: 3) biological replicates. Two-way ANOVA followed by Šídák multiple comparisons. Error bar represents the SEM. (**d**) MEA measurements on symNs derived from iPSC-FD-M1/M2/M4 still shows hyperactivity compared to hESC-ctrl-H9 (same data as in Fig. 2b for ctrl). N=(D20-H9: 9, M1: 5, M2: 8, M4: 6; D25-H9: 9, M1: 5, M2: 8, M4: 6; D30-H9: 14, M1: 5, M2: 8, M4: 3; D35-H9: 10, M1: 5, M2: 6, M4: 4; D40-H9: 7 M1: 4, M2: 7, M4: 6; D45-H9: 3, M1: 4, M2: 4, M4: 6; D50-H9: 3, M1: 3, M2: 5, M4: 6; D55-H9: 5, M1: 3,

M2: 4, M4: 7; D60-H9: 6, M1: 3, M2: 3, M4: 7) biological replicates. Two-way ANOVA followed by Šídák multiple comparisons. Error bar represents the SEM. *p<0.05, **P<0.01, ***P<0.001. (**e**) *ELP1* splicing between hESC-ctrl-H9 and iPSC-FD-S2 on day 0 and in differentiated symNs was compared by RT-qPCR. N=(D0: 3; SymN-ctrl: 4, FD: 3) biological replicates. Two-way ANOVA followed by Šídák multiple comparisons. Error bar represents the SEM. *p<0.05, ***P<0.001, ****P<0.0001. (**f**) Western blot shows that symNs from iPSC-rescued-T6 express higher levels of ELP1, but still lower than neurons from iPSC-carrier-A1. Data was quantified using one-way ANOVA followed by Tukey's multiple comparisons (right). *P<0.05. **P<0.01. N=2 biological replicates. Error bar represents the SEM. Source data are provided as a Source Data file.

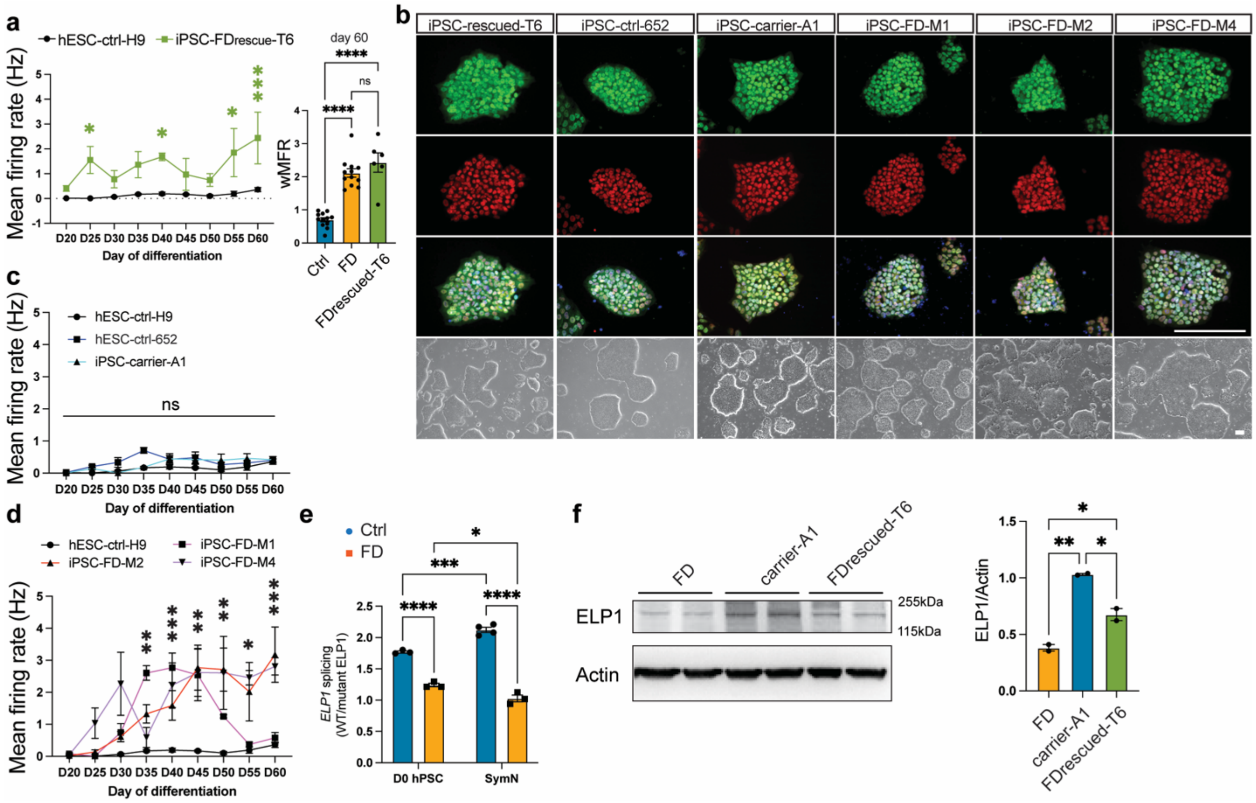

Supplementary figure 8 (related to figure 4). NGF retrograde transport is defective in Familial Dysautonomia sympathetic neurons

(**a**) Schematic illustration of compartmental symN culture using microfluidic devices. NGF medium was applied to the axon chamber on day 20. (**b**) PRPH staining (green) for the axon chambers on day 30 shows shorter distance of axon outgrowth in symNs derived from iPSC-FD-S2 compared to iPSC-ctrl-H9. Images were quantified as the bar graft on the right. N=4 biological replicates. Two-tailed Student's t-test. *P<0.05. Error bar represents the SEM. Scale bar represents 200 µm. Source data are provided as a Source Data file.

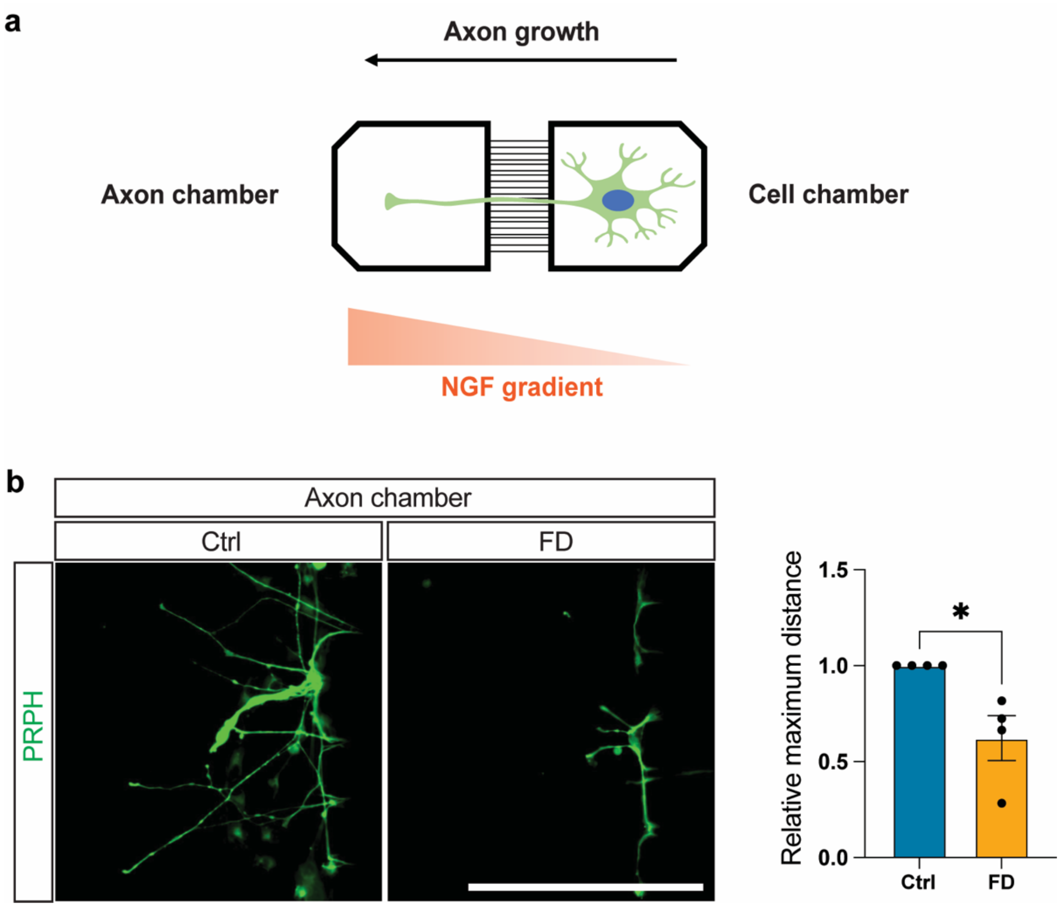

Supplementary figure 9 (related to figure 4). Bulk RNA sequencing of Familial Dysautonomia and control sympathetic neurons.

(**a**) Principal component analysis (PCA) of symNs from hESC-ctrl-H9 and iPSC-FD-S2 on day 35. (**b**) Unsupervised clustering of symNs from hESC-ctrl-H9 and iPSC-FD-S2 on day 35. (**c**) Gene ontology (GO) analysis of differentially expressed genes hESC-ctrl-H9 and iPSC-FD-S2 on day 35. Selected 20 significant annotations using False Discovery Rate (FDR<0.05) from GO biological process, GO cellular component, and KEGG pathways are shown. Adaptive FDR was calculated from the EASE Score (a modified one-tailed Fisher's Exact test from DAVID Bioinformatics Resources, see methods for details). (**d**) Z-scores of selected target genes between hESC-ctrl-H9 and iPSC-FD-S2 on day 35.

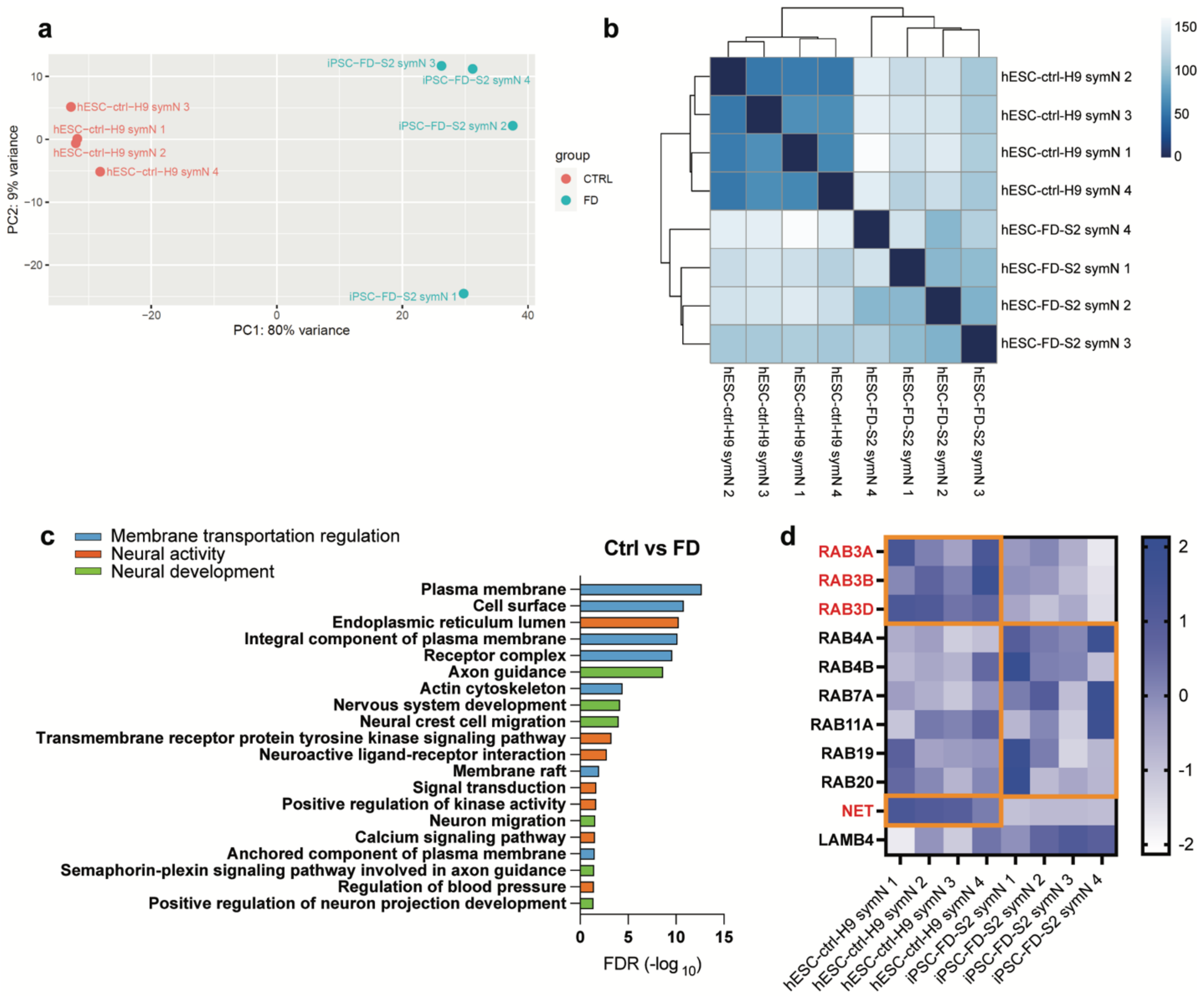
a
PC1: 80% variance
PC2: 9% variance
group
CTRL
FD
hESC-ctrl-H9 symN 1
hESC-ctrl-H9 symN 2
hESC-ctrl-H9 symN 3
hESC-ctrl-H9 symN 4
iPSC-FD-S2 symN 1
iPSC-FD-S2 symN 2
iPSC-FD-S2 symN 3
iPSC-FD-S2 symN 4
b
hESC-FD-S2 symN 1
hESC-FD-S2 symN 2
hESC-FD-S2 symN 3
hESC-FD-S2 symN 4
c
Membrane transportation regulation
Neural activity
Neural development
Ctrl vs FD
Plasma membrane
Cell surface
Endoplasmic reticulum lumen
Integral component of plasma membrane
Receptor complex
Axon guidance
Actin cytoskeleton
Nervous system development
Neural crest cell migration
Transmembrane receptor protein tyrosine kinase signaling pathway
Neuroactive ligand-receptor interaction
Membrane raft
Signal transduction
Positive regulation of kinase activity
Neuron migration
Calcium signaling pathway
Anchored component of plasma membrane
Semaphorin-plexin signaling pathway involved in axon guidance
Regulation of blood pressure
Positive regulation of neuron projection development
FDR (-log 10)
d
RAB3A
RAB3B
RAB3D
RAB4A
RAB4B
RAB7A
RAB11A
RAB19
RAB20
NET
LAMB4

Supplementary figure 10 (related to figure 5). Validation for drugs used in the mini drug screen.

Various drug conditions and treating times or dosages were tested for dexmedetomidine (**a**), clozapine (**b**), BGP-15 (**c**) and NSC-87877 (**d**). For (a), n=(25/50/60 μM: 5, 70 μM:8; each was normalized to its own vehicle control with same number) biological replicates. One-way ANOVA followed by Tukey's multiple comparisons. Error bars represent the SEM. ****P<0.0001. For (b), n=(24/48hr: 5, 72hr: 4) biological replicates. Two-way ANOVA followed by Šídák multiple comparisons. Error bar represents the SEM. **P<0.01, ***P<0.001. For (c), n=(5 min/72hr: 4, 24/48hr: 5) biological replicates. Two-way ANOVA followed by Šídák multiple comparisons. Error bar represents the SEM. For (d), n=(5 min: 5, 24/48/72hr: 4) biological replicates. Two-way ANOVA followed by Šídák multiple comparisons. Error bar represents the SEM. Data from hESC-ctrl-H9 and iPSC-ctrl-C1 are pooled as control; data from iPSC-FD-S2 and iPSC-FD-S3 are pooled as FD. Source data are provided as a Source Data file.

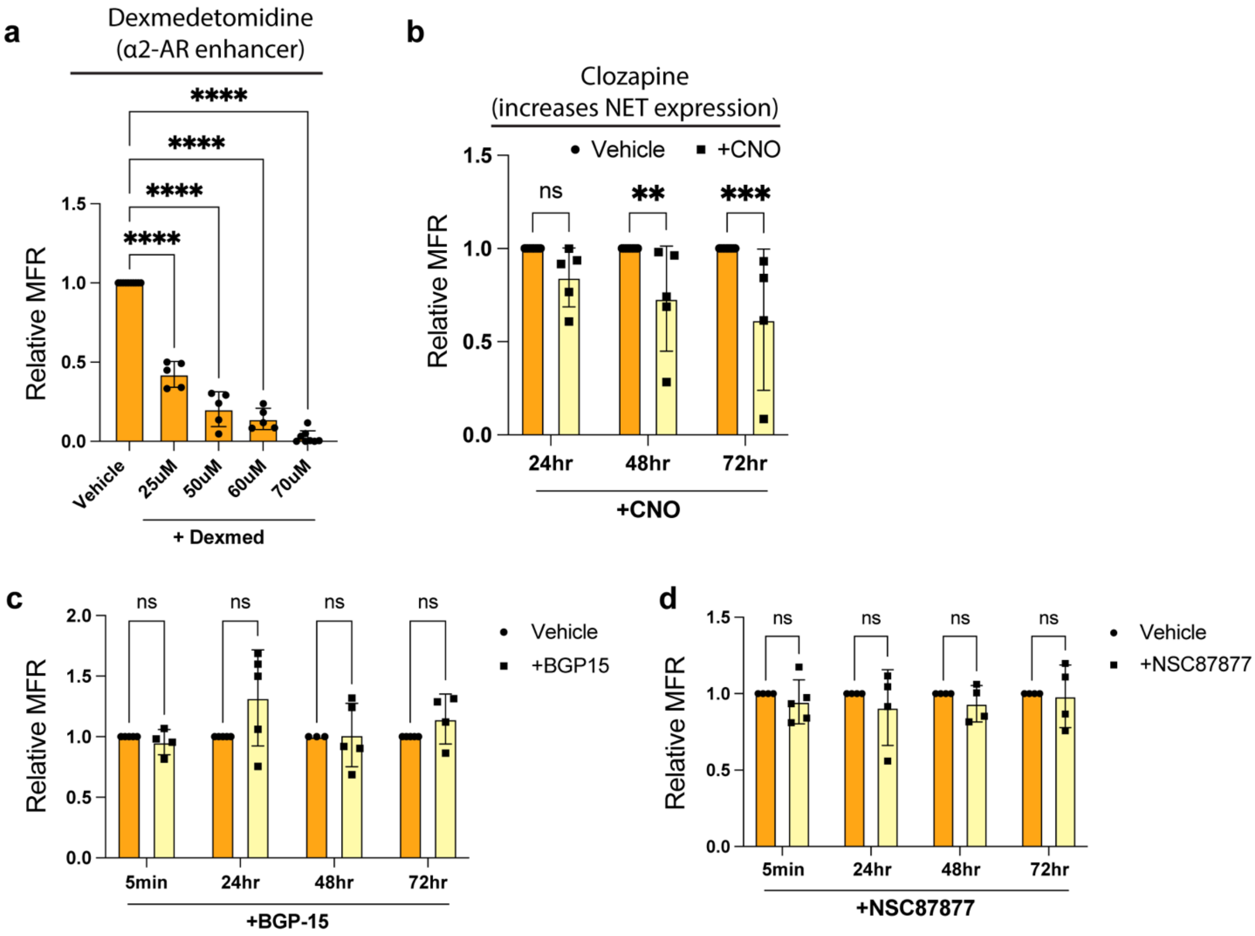

a
Dexmedetomidine
(α2-AR enhancer)
****
****
****
****
Relative MFR
1.5
1.0
0.5
0.0
Vehicle
25uM
50uM
60uM
70uM
+ Dexmed
b
Clozapine
(increases NET expression)
Vehicle
+CNO
ns
**
***
Relative MFR
1.5
1.0
0.5
0.0
24hr
48hr
72hr
+CNO
c
ns
ns
ns
ns
Relative MFR
2.0
1.5
1.0
0.5
0.0
5min
24hr
48hr
72hr
+BGP-15
Vehicle
+BGP15
d
ns
ns
ns
ns
Relative MFR
1.5
1.0
0.5
0.0
5min
24hr
48hr
72hr
+NSC87877
Vehicle
+NSC87877

Supplementary tables

Sup. Table 1. Primers used in this study

Primers	Target	Catelog/ Reference	Method	Sequence	
AAAD	Human	Origene (NM_000790)	SYBR	Forward	GGACCACAACATGCTGCTCCTT
				Reverse	CTCCACTCCATTCAGAAGGTGC
ADRA2A	Human	Origene (NM_000681)	SYBR	Forward	CTTCTGGTTCGGCTACTGCAAC
				Reverse	GGAAACCTCACACGATCCGCTT
ADRB2	Human	Origene (NM_000024)	SYBR	Forward	TACCAGAGCCTGCTGACCAAGA
				Reverse	AGTCACAGCAGGTCTCATTGGC
AP2a	Human	Origene (NM_003220)	SYBR	Forward	GACCTCTCGATCCACTCCTTAC
				Reverse	GAGACGGCATTGCTGTTGGACT
Ascl1	Human	Origene (NM_004316)	SYBR	Forward	TCTCATCCTACTCGTCGGACGA
				Reverse	CTGCTTCCAAAGTCCATTCGCAC
BRN3A	Human	Origene (NM_006237)	SYBR	Forward	AGTACCCGTCGCTGCACTCCA
				Reverse	TTGCCCTGGGACACGGCGATG
BryT	Human	Origene (NM_003181)	SYBR	Forward	CCTTCAGCAAAGTCAAGCTCACC
				Reverse	TGAACTGGGTCTCAGGGAAGCA
Ca$_v$2.1	Human	Origene (001127222)	SYBR	Forward	CTGGTAGCCTTTGCCTTCACTG
				Reverse	ACACAGCCTTGAGCTTTGGCAG
Ca$_v$2.2	Human	Origene (000718)	SYBR	Forward	TGGACCTGGAAAGCCAAGCAGA
				Reverse	GGTCACGATGTAGTGGCAGAAG
Ca$_v$1.2	Human	Origene (000719)	SYBR	Forward	GCAGGAGTACAAGAACTGTGAGC
				Reverse	CGAAGTAGGTGGAGTTGACCAC
Ca$_v$1.3	Human	Origene (000720)	SYBR	Forward	CTTCGACAACGTCCTCTCTGCT
				Reverse	GCCGATGTTCTCTCCATTCGAG
CD49d	Human	Origene (NM_000885)	SYBR	Forward	GCATACAGGTGTCCAGCAGAGA
				Reverse	AGGACCAAGGTGGTAAGCAGCT
ChAT	Human	Home-made	SYBR	Forward	GCCTTCTACAGGCTCCATCG
				Reverse	GCTCTCACAAAAGCCAGTGC
CHRNA3	Human	Origene (NM_000743)	SYBR	Forward	TGGAGACCAACCTGTGGCTCAA
				Reverse	CAGCACAATGTCTGGCTTCCAG

CHRNB4	Human	Origene (NM_000750)	SYBR	Forward	ATCTGGTTGCCTGACATCGTGC
				Reverse	TTGCAGGCGCTCTTGTAGATGG
CRHR1	Human	Origene (004382)	SYBR	Forward	TGCCAGGAGATCCTCAATGAGG
				Reverse	AGCCGCAGAAAGAGGACAAAGG
CRHR2	Human	Origene (001883)	SYBR	Forward	GGTGGACTACATCTACCAAGGC
				Reverse	GATTGTCTCGGATGTGGTGGAC
cTnT	Human	Origene (000364)	SYBR	Forward	AAGAGGCAGACTGAGCGGGAAA
				Reverse	AGATGCTCTGCCACAGCTCCTT
DBH	Human	Origene (NM_000787)	SYBR	Forward	GCCTTCATCCTCACTGGCTACT
				Reverse	CAGCACTGTGACCACCTTTCTC
Eya1	Human	Origene (172058)	SYBR	Forward	TGGCATCACCAGCCAAGCAGTT
				Reverse	CCATCTGAACCTCGACGCAATC
ELP1_WT		Home-made	SYBR	Forward	GCAGCAATCATGTGTCCCA
				Reverse	ACCAGGGCTCGATGATGAA
ELP1_mutant		Home-made	SYBR	Forward	CACAAAGCTTGTATTACAGACT
				Reverse	GAAGTTTCCACATTTCCAAG
GA$_A$R	Human	Home-made	SYBR	Forward	AGGAAGGCTTTTCGGCATCT
				Reverse	GGGATCGTTCACACTCTGGG
GAPDH	Human	Origene (NM_002046)	SYBR	Forward	GTCTCCTCTGACTTCAACAGCG
				Reverse	ACCACCCTGTTGCTGTAGCCAA
GAP43	Human	Origene (002045)	SYBR	Forward	GAGCAGCCAAGCTGAAGAGAAC
				Reverse	GCCATTTCTTAGAGTTCAGGCATG
GATA3	Human	Origene (NM_001002295)	SYBR	Forward	ACCACAACCACACTCTGGAGGA
				Reverse	TCGGTTTCTGGTCTGGATGCCT
HOXB2	Human	Home-made	SYBR	Forward	CAATCCGCCACGTCTCCTTC
				Reverse	CCAGGCCATCTGCAGGC
HOXB5	Human	Home-made	SYBR	Forward	CGAAATAGACGAGGCCAGCG
				Reverse	CGGCCCGGTCATATCATGG
HOXB7	Human	Home-made	SYBR	Forward	CGCCCTTTGAGCAGAACCTC
				Reverse	CGTTTGCGGTCAGTTCCTGAG
HOXC9	Human	Frith et al. (2018)	SYBR	Forward	GCAGCAAGCACAAAGAGGA
				Reverse	CGTCTGGTACTTGGTGTAGGG
HOXA10	Human	Home-made	SYBR	Forward	GCGAGCCCTCGATTCGC
				Reverse	GAATTGCCCAGGGAATCCTTCTC
HAND2	Human	Origene (NM_02197)	SYBR	Forward	GGCAGAGATCAAGAAGACCGAC
				Reverse	CGGCCTTTGGTTTTCTTGTCGTT

KCNQ2	Human	Origene (172107)	SYBR	Forward	TCATCGGTGTCTCCTTCTTCGC
				Reverse	GAGAGGTTGGTGGCGTAGAATC
KCNQ3	Human	Origene (004519)	SYBR	Forward	CGTCTGATTGCCGCCACCTTTT
				Reverse	TTCTGACGGTGTTGCTCCTGCA
MAOA	Human	Origene (NM_000240)	SYBR	Forward	TCTGAGCCTCACGAAGTGTCTG
				Reverse	ATCCGTTCGCTCACTTGACCAG
MAP2	Human	Origene (002374)	SYBR	Forward	AGGCTGTAGCAGTCCTGAAAGG
				Reverse	CTTCCTCCACTGTGACAGTCTG
Maxi-K	Human	Origene (002247)	SYBR	Forward	TATCTCTCCAGTGCCTTCGTGG
				Reverse	CTCTCTCGGTTGGCAGACTTGT
$Na_v1.7$	Human	Origene (002977)	SYBR	Forward	GTGGAAGGATTGTCAGTTCTGCG
				Reverse	GCCAACACTAAGGTGAGGTTACC
NKX2.5	Human	Origene (004387)	SYBR	Forward	AAGTGTGCGTCTGCCTTTCCCG
				Reverse	TTGTCCGCCTCTGTCTTCTCCA
NPY	Human	Origene (000905)	SYBR	Forward	GCTGCGACACTACATCAACCTC
				Reverse	CTGTGCTTTCTCTCATCAAGAGG
Oct4	Human	Origene (NM_002701)	SYBR	Forward	CCTGAAGCAGAAGAGGATCACC
				Reverse	AAAGCGGCAGATGGTCGTTTGG
OTX2	Human	Origene (021728)	SYBR	Forward	GGAAGCACTGTTTGCCAAGACC
				Reverse	CTGTTGTTGGCGGCACTTAGCT
PAX6	Human	Origene (001604)	SYBR	Forward	CTGAGGAATCAGAGAAGACAGGC
				Reverse	ATGGAGCCAGATGTGAAGGAGG
PHOX2B	Human	Frith et al. (2018)	SYBR	Forward	CTACCCCGACATCTACACTCG
				Reverse	CTCCTGCTTGCGAAACTTG
PRPH	Human	Home-made	SYBR	Forward	GTGCCCGTCCATTCTTTTGC
				Reverse	GTCACCACCTCCCCATTCCG
P2RX4	Human	Origene (002560)	SYBR	Forward	GTGGCGGATTATGTGATACCAGC
				Reverse	CACACAGTGGTCGCATCTGGAA
P2RX7	Human	Origene (002562)	SYBR	Forward	CGACTAGGAGACATCTTCCGAG
				Reverse	GCAGTGATGGAACCAACGGTCT
RAB3A	Human	Origene (NM_002866)	SYBR	Forward	CGCTATGCTGACGACTCGTTCA

				Reverse	GGTAGTATGCGGTGGTGATGGT
RAB4A	Human	Origene (NM_004578)	SYBR	Forward	ATGCAGGAACTGGCAAATCTTGC
				Reverse	CGTTCTTGTCCTGCTGTATCCC
RAB7A	Human	Origene (NM_004637)	SYBR	Forward	GTGATGGTGGATGACAGGCTAG
				Reverse	AGTCTGCACCTCTGTAGAAGGC
RAB11A	Human	Origene (NM_004663)	SYBR	Forward	AGCACCATTGGAGTAGAGTTTGC
				Reverse	AAGGCACCTACAGCTCCACGAT
SIX1	Human	Origene (005982)	SYBR	Forward	AGGTCAGCAACTGGTTTAAGAACC
				Reverse	GAGGAGAGAGTTGGTTCTGCTTG
SLC6A2	Human	Origene (NM_001043)	SYBR	Forward	CAGGTTCAGCAACGACATCCAG
				Reverse	GTCGTAGGTGAGTGGCTTGAAG
SOX10	Human	Home-made	SYBR	Forward	CCAGGCCCACTACAAGAGC
				Reverse	CTCTGGCCTGAGGGGTGC
SOX17	Human	Origene (022454)	SYBR	Forward	ACGCTTTCATGGTGTGGGCTAAG
				Reverse	GTCAGCGCCTTCCACGACTTG
Synaptophysin	Human	Origene (003179)	SYBR	Forward	TCGGCTTTGTGAAGGTGCTGCA
				Reverse	TCACTCTCGGTCTTGTTGGCAC
TH	Human	Origene (NM_199292)	SYBR	Forward	GCTGGACAAGTGTCATCACCTG
				Reverse	CCTGTACTGGAAGGCGATCTCA
VIP	Human	Origene (NM_003381)	SYBR	Forward	CCAGTCAAACGTCACTCAGATGC
				Reverse	CTGGAAAGTCGGGAGATTCTCC
VMAT1	Human	Origene (NM_003053)	SYBR	Forward	CAGCCTTCCAAAGTCTCTCCTG
				Reverse	GCACATGGTCTGCATCATCCAG
HAND2	Mouse	Origene (010402)	SYBR	Forward	ATCGCCTACCTCATGGATCTGC
				Reverse	CTGCTCACTGTGCTTTTCAAGATC
PHOX2B	Mouse	Origene (008888)	SYBR	Forward	CCCGATAAGGACCACTTTTGGG
				Reverse	CCGTGGTCGGTGAAGAGTTTGT
ASCL1	Mouse	Origene (008553)	SYBR	Forward	CGGAACTGATGCGCTGCAAACG
				Reverse	GGCAAAACCCAGGTTGACCAAC
TH	Mouse	Origene (009377)	SYBR	Forward	TGCACACAGTACATCCGTCATGC
				Reverse	GCAAATGTGCGGTCAGCCAACA

PRPH	Mouse	Origene (013639)	SYBR	Forward	CGAGATAGCCACCTACAGGAAG
				Reverse	TCTTGCTGTGGCTATCCTGGAG
GAPDH	Mouse	Origene (008084)	SYBR	Forward	CATCACTGCCACCCAGAAGACTG
				Reverse	ATGCCAGTGAGCTTCCCGTTCAG
**Primers**	**Target**	**Catelog/Reference**	**Method**	**Sequence**	

Sup. Table 2. Antibodies used in this study

Antibodies	Brand	Catalog	Host	Dilution	Clone	LOT#
AP2a	Abcam	ab108311	Rabbit	1:400	EPR2688(2	GR3246539-1
Ascl1	BD Pharmingen	556604	Mouse IgG1	1:200	24B72D11.1	Not available
α2AR	Abcam	ab85570	Rabbit	1:200	Not available	Not available
α-actinin	Sigma	A7811	Mouse IgG1	1:1000	EA-53	0000097265
β2AR	Santa Cruz Biotechnology	SC-271322	Mouse IgG2b	1:200	Not available	L0219
CD49D	BioLegend	304313	Mouse IgG1	5 µl/million cells in 100 µl volume	9F10	Not available
c-Fos	Santa Cruz Biotechnology	sc-166940	Mouse IgG1	1:1000 for WB	Not available	K3020
ChAT	Millipore	AB144P	Goat	1:100	NG1780580	3491643
DAPI	Sigma	D9542	-	1:1000	-	Not available
ELP1	Boster Bio	A31687	Rabbit	1:50 (IF) and 1:1000 (WB)	Not available	23371104
GFP	Abcam	ab13970	Chicken	1:1000	Not available	Not available
HOXC9	Abcam	ab50839	Mouse	1:100	HOXCA6E6	GR3239200-2
Ki67	Abcam	ab15580	Rabbit	1:1000	Not available	GR3452679-1
MAP2	Novus Biologicals	NB600-1372	Mouse IgG1	1:200	AP20	Not available
NANOG	cell signaling	C734G	Rabbit	1:400	D73G4	KKJ0617121
Norepinephrine	Abcam	ab8887	Rabbit	1:500	Not available	GR3361983-2
anti-human NET	Mab	NET17-1	Mouse	1:1000 for IF/WB	3-6C1	Not available
anti-mouse NET	Mab	NET05-2	Mouse	1:500	2-3B2	Not available
OCT4	Santa Cruz Biotechnology	sc-5279 lotD2211	mIgG2a	1:200	Not available	Not available
PRPH	Santa Cruz Biotechnology	SC-377093/H0112	Mouse IgG2a	1:200	Not available	Not available
Rab3A	Abcam	ab3335	Rabbit	1:500	Not available	GR3416912-1
SOX10	Santa Cruz Biotechnology	sc-365692	Mouse IgG1	1:100	Not available	Not available
TH	Pel-Freez	P40101- 150	Rabbit	1:500	Not available	aj03190
TUJ1	Biolegend	802001	Rabbit	1:1500	Poly18020	B259312

	Biolegend	801201	Mouse IgG2a	1:1500	TUJ1	B209227

Suppl Table 3. Cell lines used in this study

Name	hyperactivity phenotype	*ELP1* genotype	*LAMB4* genotype	Coriell fibroblast number
hESC-ctrl-H9	physiological	+/+	+/+	-
iPSC-ctrl-C1	physiological	+/+	+/+	AG02602
iPSC-ctrl-652	physiological	+/+	+/+	GM01652
iPSC-FDrescHet-T6	hyperactive	+/-	+/-	GM04899
iPSC-carrierHet-A1	physiological	+/-	+/-	GM04895
iPSC-FD-mild-M1	hyperactive	-/-	+/+	GM02341
iPSC-FD-mild-M2	hyperactive	-/-	+/+	GM02342
iPSC-FD-mild-M4	hyperactive	-/-	+/+	GM04663
iPSC-FD-severe-S2	hyperactive	-/-	+/-	GM04899
iPSC-FD-severe-S3	hyperactive	-/-	+/-	GM04589
