# Supplementary material for: Norepinephrine transporter defects lead to sympathetic hyperactivity in Familial Dysautonomia models
Source: Nat Commun. 2022 Nov 17;13:7032. doi: 10.1038/s41467-022-34811-7 (PMC9671909; doi:10.1038/s41467-022-34811-7)
Supplement: Supplementary file 2 — Reporting Summary [file 41467_2022_34811_MOESM2_ESM.pdf]

nature portfolio

Corresponding author(s): Nadja Zeltner

Last updated by author(s): 10/26/2022

Reporting Summary

Nature Portfolio wishes to improve the reproducibility of the work that we publish. This form provides structure for consistency and transparency in reporting. For further information on Nature Portfolio policies, see our Editorial Policies and the Editorial Policy Checklist.

Statistics

For all statistical analyses, confirm that the following items are present in the figure legend, table legend, main text, or Methods section.

n/a	Confirmed	
☐	☒	The exact sample size (n) for each experimental group/condition, given as a discrete number and unit of measurement
☐	☒	A statement on whether measurements were taken from distinct samples or whether the same sample was measured repeatedly
☐	☒	The statistical test(s) used AND whether they are one- or two-sided<br>*Only common tests should be described solely by name; describe more complex techniques in the Methods section.*
☒	☐	A description of all covariates tested
☐	☒	A description of any assumptions or corrections, such as tests of normality and adjustment for multiple comparisons
☐	☒	A full description of the statistical parameters including central tendency (e.g. means) or other basic estimates (e.g. regression coefficient) AND variation (e.g. standard deviation) or associated estimates of uncertainty (e.g. confidence intervals)
☐	☒	For null hypothesis testing, the test statistic (e.g. F, t, r) with confidence intervals, effect sizes, degrees of freedom and P value noted<br>*Give P values as exact values whenever suitable.*
☒	☐	For Bayesian analysis, information on the choice of priors and Markov chain Monte Carlo settings
☒	☐	For hierarchical and complex designs, identification of the appropriate level for tests and full reporting of outcomes
☒	☐	Estimates of effect sizes (e.g. Cohen's d, Pearson's r), indicating how they were calculated

Our web collection on statistics for biologists contains articles on many of the points above.

Software and code

Policy information about availability of computer code

Data collection	LAS X, GEN5 3.05, Axion AxIS Navigator, Axion Neural metric tool, iBright Imaging system, CFX96 Real time System, CytExpert 2.0,
Data analysis	Prism 9, Fiji (ImageJ), CFX Maestro, FlowJo10.5.3, BaseSpace , Galaxy web platform (usegalaxy.org), FastQC (version 0.11.9)(http://www.bioinformatics.babraham.ac.uk/projects/fastqc/), Trimmomatic (version 0.38), HISAT2 (version 2.21), QualiMap BamQC (version 2.2.2c), HTseq (version 0.91), DESeq2 (version 1.34.0), DAVID functional annotation tool (https://david.ncifcrf.gov/).

For manuscripts utilizing custom algorithms or software that are central to the research but not yet described in published literature, software must be made available to editors and reviewers. We strongly encourage code deposition in a community repository (e.g. GitHub). See the Nature Portfolio guidelines for submitting code & software for further information.

Data

Policy information about availability of data

All manuscripts must include a data availability statement. This statement should provide the following information, where applicable:

- Accession codes, unique identifiers, or web links for publicly available datasets
- A description of any restrictions on data availability
- For clinical datasets or third party data, please ensure that the statement adheres to our policy

All data generated or analyzed in this study are included in this published article and its supplementary data file. Raw data points and uncut western blot gel data generated in this study have been deposited in the figshare.com database under accession code: DOI 10.6084/m9.figshare.21394737. FASTQ files for RNA sequencing data was deposited through NCBI Gene Expression Omnibus, accession number GSE212255. Source data are provided with this paper.

Field-specific reporting

Please select the one below that is the best fit for your research. If you are not sure, read the appropriate sections before making your selection.

- [x] Life sciences
- [ ] Behavioural & social sciences
- [ ] Ecological, evolutionary & environmental sciences

For a reference copy of the document with all sections, see nature.com/documents/nr-reporting-summary-flat.pdf

Life sciences study design

All studies must disclose on these points even when the disclosure is negative.

Sample size	Sample sizes were a minimum of three biological repeats (for in vitro differentiations this is defined as independent differentiations started on different days from consecutive splits or from freshly thawed cells) or experimental animals. However, in most experiments more than 3 biological repeats were assessed, mostly when the distribution/variation between data points was large. See Figure legends and methods.
Data exclusions	Data were excluded when the values were out of the range of two standard deviations (SD). This criteria was decided before the data was acquired.
Replication	In our previous work (Wu et al., 2020 JOVE) we established checkpoints to assess the quality of individual differentiations. Here, we followed these checkpoints for each differentiation and the replicate was abandoned if one or more checkpoints were not passed properly. Under these circumstances all results in this work were reproducible.
Randomization	Randomization was not applied to this work, since there are no drug treatments or animals/conditions that were tested against each other.
Blinding	The investigators were not blinded, as blinding was not possible or feasible due to the experimental designs, lack on human power and resources.

Reporting for specific materials, systems and methods

We require information from authors about some types of materials, experimental systems and methods used in many studies. Here, indicate whether each material, system or method listed is relevant to your study. If you are not sure if a list item applies to your research, read the appropriate section before selecting a response.

Materials & experimental systems

n/a	Involved in the study	
[ ]	[x]	Antibodies
[ ]	[x]	Eukaryotic cell lines
[x]	[ ]	Palaeontology and archaeology
[ ]	[x]	Animals and other organisms
[x]	[ ]	Human research participants
[x]	[ ]	Clinical data
[x]	[ ]	Dual use research of concern

Methods

n/a	Involved in the study	
[x]	[ ]	ChIP-seq
[ ]	[x]	Flow cytometry
[x]	[ ]	MRI-based neuroimaging

Antibodies

Antibodies used

See Suppl. table 2:
Antibodies Brand Catalog Host Dilution Clone LOT#
AP2a Abcam ab108311 Rabbit 1:400 EPR2688(2 GR3246539-1
Ascl1 BD Pharmingen 556604 Mouse IgG1 1:200 24B72D11.1 Not available
α2AR Abcam ab85570 Rabbit 1:200 Not available Not available
α-actinin Sigma A7811 Mouse IgG1 1:1000 EA-53 0000097265
β2AR Santa Cruz Biotechnology SC-271322 Mouse IgG2b 1:200 Not available L0219
CD49D BioLegend 304313 Mouse IgG1 5 µl/million cells in 100 µl volume 9F10 Not available
c-Fos Santa Cruz Biotechnology sc-166940
Mouse IgG1 1:1000 for WB Not available K3020
ChAT Millipore AB144P Goat 1:100 NG1780580 3491643
DAPI Sigma D9542 - 1:1000 - Not available
ELP1 Boster Bio A31687 Rabbit 1:50 (IF) and 1:1000 (WB) Not available 23371104
GFP Abcam ab13970 Chicken 1:1000 Not available Not available
HOXC9 Abcam ab50839 Mouse 1:100 HOXCA6E6 GR3239200-2
Ki67 Abcam ab15580 Rabbit 1:1000 Not available GR3452679-1
MAP2 Novus Biologicals NB600-1372 Mouse IgG1 1:200 AP20 Not available
NANOG cell signaling C734G Rabbit 1:400 D73G4 KKJ0617121

Norepinephrine Abcam ab8887 Rabbit 1:500 Not available GR3361983-2
anti-human NET Mab NET17-1 Mouse 1:1000 for IF/WB 3-6C1 Not available
anti-mouse NET Mab NET05-2 Mouse 1:500 2-3B2 Not available
OCT4 Santa Cruz Biotechnology sc-5279 lotD2211 mIgG2a 1:200 Not available Not available
PRPH Santa Cruz Biotechnology SC-377093/H0112 Mouse IgG2a 1:200 Not available Not available
Rab3A Abcam ab3335 Rabbit 1:500 Not available GR3416912-1
SOX10 Santa Cruz Biotechnology sc-365692 Mouse IgG1 1:100 Not available Not available
TH Pel-Freez P40101- 150 Rabbit 1:500 Not available aj03190
TUJ1 Biolegend 802001 Rabbit 1:1500 Poly18020 B259312
Biolegend 801201 Mouse IgG2a 1:1500 TUJ1 B209227

Validation

Antibody validation
Antibody name reactivity applicability
AP2a reactive to Mouse, Rat, Human applicable for ICC/IF, WB, IHC-P, Flow Cyt (Intra), IP, Knockout validated
Ascl1 reactive to African green monkey, human, mouse, rat, rabbit applicable for WB (Routinely Tested), IHC (reported)
α2AR reactive to Human applicable for WB, IP, IF, IHC-P, ELISA, 4 reported references
α-actinin reactive to fish, snake, frog, goat, hamster, pig, canine, mouse, feline, chicken, lizard, bovine, human, sheep, rat, rabbit applicable for WB, IHC/IF, ELISA, 6 reported references
β2AR reactive to Human applicable for WB, IP, IF, IHC(P) and ELISA, 8 reported references
CD49D reactive to Human, African Green, Baboon, Cat (Feline), Cattle (Bovine, Cow), Chimpanzee, Common Marmoset, Cynomolgus, Dog (Canine), Horse (Equine), Rhesus, Sheep (Ovine), Squirrel Monkey applicable for flow cytometry, 9 reported references
ChAT reactive to Avian, chicken, guinea pig, monkey, rat, and opposum applicable for immunohistochemical staining, western blotting, immunocytochemistry
c-Fos reactive to Mouse, Rat, Human applicable for IHC-P, WB, ICC/IF, 8 reported references
ELP1 reactive to Human, Mouse applicable for ELISA, IF, ICC, WB, validated with known positive and negative samples to ensure specificity and high affinity
GFP Species independent applicable for WB, ICC/IF, 2684 reported references
HOXC9 reactive to Human applicable for ICC/IF, WB, 7 reported references
Ki67 reactive to Human, Mouse applicable for IHC-P, ICC, Knockout validated
MAP2 reactive to Hu, Mu, Rt, Ca, Pgn, Xp applicable for WB, ICC/IF, IHC, IHC-P, 10 reported references
NANOG reactive to H-Human M-Mouse R-Rat Hm-Hamster Mk-Monkey Vir-Virus Mi-Mink C-Chicken Dm-D. melanogaster X-Xenopus Z-Zebrafish B-Bovine Dg-Dog Pg-Pig Sc-S. cerevisiae Ce-C. elegans Hr-Horse applicable for WB, IP, IHC, ChIP, IF, Flow Cytometry, ELISA, 336 reported references
Norepinephrine Species independent applicable for ICC, IHC-Fr, IHC-P
hNET1 reactive to Human and non-human primate WB, IHC applicable for WB, IHC, 25 reported references
mNET1 reactive to Human and non-human primate WB, IHC applicable for WB, IHC, 5 reported references
OCT4 reactive to mouse, rat and human applicable for WB, IP, IF, IHC-P, Flow Cytometry and ELISA
PRPH reactive to mouse, rat and human applicable for WB, IP, IF, IHC-P and ELISA
Rab3a reactive to mouse, Rat, Human applicable for WB, IHC, IF, 14 reported references
SOX10 reactive to human, mouse, rat, canine, bovine and porcine applicable for WB, IP, IF, IHC-P and ELISA, 67 reported references
TH reactive to Mammalian, Non-Mammalian applicable for WB, IHC, IF, 6 reported references
TUJ1 802001 reactive to Human, Mouse, Rat applicable for WB, IHC, IF, 100 reported references
801201 reactive to Human, Mouse, Rat applicable for WB, IHC, IF, 553 reported references

Eukaryotic cell lines

Policy information about cell lines

Cell line source(s)

hPSC lines: hES-ctrl-H9=WiCell WA09; iPSC-ctrl-C1, iPSC-FD-S2, iPSC-FD-S3, iPSC-rescued-T6, iPSC-carrier-A1, iPSC-FD-M1, iPSC-FD-M2 were characterized in Zeltner, 20161. iPSC-ctrl-652 were reprogrammed from fibroblasts from a 11-year old, female, healthy donor, Coriell #GM01652, iPSC-FD-M4 were reprogrammed from fibroblasts from a 2-year old female patient with FD, Coriell #GM04663, see table 3:
Suppl Table 3

Name hyperactivity phenotype ELP1 genotype LAMB4 genotype Coriell fibroblast number
hESC-ctrl-H9 physiological +/+ +/+ -
iPSC-ctrl-C1 physiological +/+ +/+ AG02602
iPSC-ctrl-652 physiological +/+ +/+ GM01652

iPSC-FDrescHet-T6 hyperactive +/- +/- GM04899
iPSC-carrierHet-A1 physiological +/- +/- GM04895

iPSC-FD-mild-M1 hyperactive -/- +/+ GM02341
iPSC-FD-mild-M2 hyperactive -/- +/+ GM02342
iPSC-FD-mild-M4 hyperactive -/- +/+ GM04663

iPSC-FD-severe-S2 hyperactive -/- +/- GM04899

	iPSC-FD-severe-S3 hyperactive -/- +/- GM04589
Authentication	The authentification of the following cell lines for our purposes was published earlier in Zeltner et al. 2016: iPSC-ctrl-C1, iPSC-FD-S2, iPSC-FD-S3, iPSC-rescued-T6, iPSC-carrier-A1, iPSC-FD-M1, iPSC-FD-M2. The remaining cell lines were authenticated in this work, see sup. fig 3 for morphology and pluripotency.
Mycoplasma contamination	All cell lines are routinely tested to be mycoplasma negative every two weeks.
Commonly misidentified lines (See ICLAC register)	n/a

Animals and other organisms

Policy information about studies involving animals; ARRIVE guidelines recommended for reporting animal research

Laboratory animals	SOX10-cre;Elp1Loxp/LoxP CKO C57BL/6 mice were provided by Dr. Hong-Xiang Liu's lab at University of Georgia and wnt1-cre;Elp1Loxp/LoxP CKO C57BL/6 mice by Dr. Frances Lefcort's lab at Montana State University. Embryos were sacrificed at E14.5 and the sex of embryos was not tested or assessed in this study.
Wild animals	no wild animals were used.
Field-collected samples	n/a
Ethics oversight	Animal protocol IACUC #2021-35-81 (Lefcort, Montana State University, Department of microbiology and immunology), #A2019 05-013 (Liu, University of Georgia, Animal Dairy sciences).

Note that full information on the approval of the study protocol must also be provided in the manuscript.

Flow Cytometry

Plots

Confirm that:

- [x] The axis labels state the marker and fluorochrome used (e.g. CD4-FITC).
- [x] The axis scales are clearly visible. Include numbers along axes only for bottom left plot of group (a 'group' is an analysis of identical markers).
- [x] All plots are contour plots with outliers or pseudocolor plots.
- [x] A numerical value for number of cells or percentage (with statistics) is provided.

Methodology

Sample preparation	Day 10 NCCs were washed with 1x PBS and dissociated in accutase. The cell/accutase mixture was washed by mixing the solution with FACS buffer that contains 1x DMEM (Life Technologies, 10829-018), 2% FBS (Atlanta Biologicals, S11150) and 200 mM L-Glutamine. The cells were spun twice at 200 g for 4 min. Count the cells and incubate 1x106 cells with mouse anti-CD49d-PECy7 (Biolegend, 304313, 1:20 in 100µl) for 20 min on ice. After incubation, wash cells by mixing the mixture with FACS buffer and spinning twice at 200 g for 4 min.
Instrument	Beckman Coulter CytoFLEX
Software	FLowJo 10.5.3
Cell population abundance	In this manuscript, the sorting process was used for cell population determination, and no cells were maintained after the process. However, in our previous publication (Wu et al. 2020, JOVE) we showed that about half of the CD49d+ cells can be harvested after sorting. We found that only CD49d+ cells can survive the suspension culture environment and form nice and solid, smooth neural crest spheroids.
Gating strategy	The gating strategy is illustrated in Sup. Fig 1a and Fig 1d and was based on unstained control cells. The area of the solid CD49d+ cluster was gated.

- [x] Tick this box to confirm that a figure exemplifying the gating strategy is provided in the Supplementary Information.
