# Supplementary material for: Norepinephrine transporter defects lead to sympathetic hyperactivity in Familial Dysautonomia models
Source: Nat Commun. 2022 Nov 17;13:7032. doi: 10.1038/s41467-022-34811-7 (PMC9671909; doi:10.1038/s41467-022-34811-7)
Supplement: Supplementary file 4 — Description of Additional Supplementary Files [file 41467_2022_34811_MOESM4_ESM.pdf]

## Description of Additional Supplementary Files

**Supplementary Movie 1**: Beating hPCS-derived cardiomyocytes at day 7 of differentiation.
